# Heterocycles [*h*]-Fused Onto 4-Oxoquinoline-3-Carboxylic Acid, Part VIII [1]. Convenient Synthesis and Antimicrobial Properties of Substituted Hexahydro[1,4]diazepino[2,3-*h*]quinoline-9-carboxylic acid and Its Tetrahydroquino[7,8-*b*]benzodiazepine Analog

**DOI:** 10.3390/molecules13112880

**Published:** 2008-11-18

**Authors:** Yusuf M. Al-Hiari, Rana Abu-Dahab, Mustafa M. El-Abadelah

**Affiliations:** 1 Department of Pharmaceutical Sciences, Faculty of Pharmacy, The University of Jordan, Amman-11942, Jordan; 2 Department of Biopharmaceutics and Clinical Pharmacy, Faculty of Pharmacy, The University of Jordan, Amman-11942, Jordan; E-mail: abudahab@ju.edu.jo (R. A-D.); 3 Department of Chemistry, Faculty of Science, The University of Jordan, Amman-11942, Jordan; E-mail: mustelab@ju.edu.jo (M. E-A.)

**Keywords:** 7-Chloro-8-nitro-4-oxoquinoline-3-carboxylic acid, *β*-alanine, diazepino[2,3-*h*]quinoline, 2-aminobenzoic acid, quino[7,8-*b*]benzodiazepine, S_N_-Ar reaction, antibacterial activity.

## Abstract

[1,4]Diazepino[2,3-*h*]quinolone carboxylic acid **3** and its benzo-homolog tetrahydroquino[7,8-*b*]benzodiazepine-3-carboxylic acid **5 **were prepared *via* PPA-catalyzed thermal lactamization of the respective 8-amino-7-substituted-1,4-dihydro-quinoline-3-carboxylic acid derivatives **8**, **10**. The latter compounds were obtained by reduction of their 8-nitro-7-substituted-1,4-dihydroquinoline-3-carboxylic acid precursors **7**, **9** which, in turn, were prepared by reaction of 7-chloro-1-cyclopropyl-6-fluoro-8-nitro-1,4-dihydroquinoline-3-carboxylic acid (**6**) with each of *β*-alanine and anthranilic acid. All intermediates and target compounds were characterized using elemental analysis, NMR, IR and MS spectral data. The prepared targets and the intermediates have shown interesting antibacterial activity mainly against Gram positive strains. In particular, compound **8** showed good activity against *S. aureus* (MIC = 0.39 µg/mL) and *B. subtilis* (MIC = 0.78 µg/mL). Compounds **5a** and **9** have also displayed good antifungal activity against *C. albicans* (MIC = 1.56 µg/mL and 0.78 µg/mL, respectively). None of the compounds tested showed any anticancer activity against solid breast cancer cell line MCF-7 cells or a human breast adenocarcinoma cell line.

## Introduction

**Figure 1:**
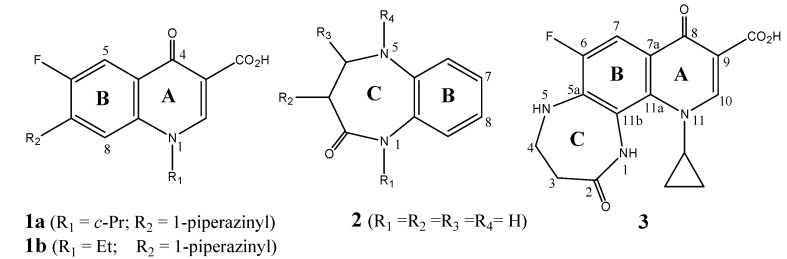
Structures of fluorquinolones **1a**, **1b**, 1,3,4,5-tetrahydro-2*H*-benzo[*b*][1,4]-diazpine-2-one (**2**) and 2,8-dioxohexahydro-1*H*-[1,4]Diazepino[2,3-*h*]quinoline carboxylic acid (**3**).

Fluoroquinolones [e.g. ciprofloxacin (**1a**) or norfloxacin (**1b**)] are successful synthetic antiinfectious agents [2,3,4,5,6,7,8,9,10,11,12,13]. On the other hand, 1,3,4,5-tetrahyro-2*H*-benzo[*b*][1,4]diazpine-2-one (**2**, Figure 1) and several substituted derivatives thereof, are of considerable interest both synthetically [14,15,16,17] and pharmacologically [18,19,20,21,22,23,24,25,26,27,28]. Depending on the nature of substituents on ring C of **2**, such derivatives exhibit antidiuretic activity [18], hyperuricemic activity [19], vasopressin and oxytocin antagonist activity [20,21], antiamoebic/antimicrobial [22], analgesic/antiinflammatory [23,24] and antitumor activities [25,26,27,28].

**Figure 2:**
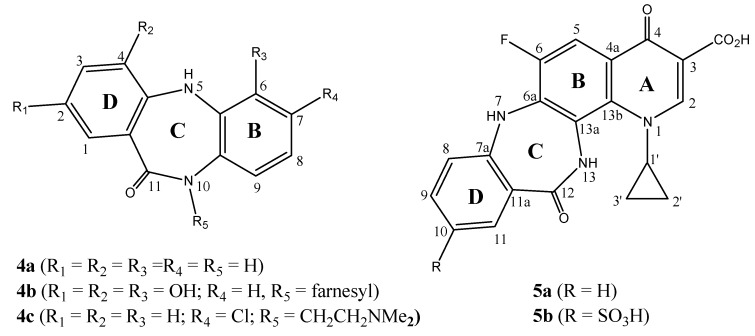
Structures of 5,10-dihydro-11*H*-dibenzo[*b*,*e*][1,4]diazepine-11-one (**4a**), diazepinomicin (**4b**), clobenzepam (**4c**) and 4-12-dioxo-tetrahyroquino[7,8-*b*][1,4]benzodiazpine-3-carboxyic acid derivatives **5a**,**b**.

The dibenzo homologs of **3 **and related derivatives, 5,10-dihydro-11*H*-dibenzo[*b*,*e*][1,4]diazepine-11-ones (e.g. **4a**, Figure 2), were prepared and reported to display different biological activities [29,30,31,32,33,34,35,36,37,38,39,40,41,42,43,44,45,46,47,48,49,50,51]. Some substituted derivatives, such as the natural antibiotic diazepinomicin (**4b**), have been isolated as dibenzodiazepine alkaloids from natural sources [29]. Other derivatives such as clobenzepam (**4c**, Figure 2), and related drugs (e.g. dibenzepine, propizepine, pirenzepine) are successful antidepressant agents [30,31,32,33]. Some of these derivatives were reported to exhibit muscarine receptor antagonist activity [34,35], antimicrobial activity [36,37,38], oxytocin and vasopressin antagonist activity [39,40], antiarrhythmic activity [41,42,43], hypoglycemic activity [44], analgesic and anti-inflammatory activity [45,46] and antitumor activity [47,48,49,50,51].

**Scheme 1:**
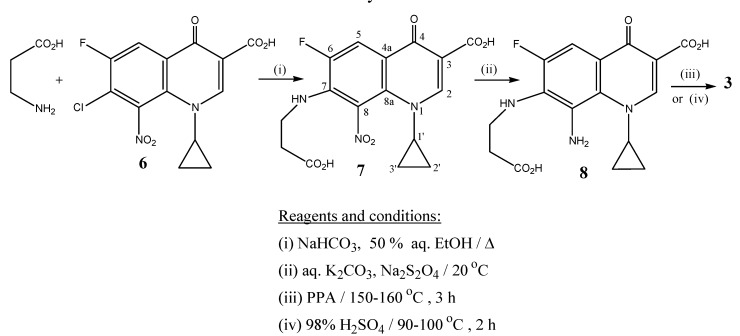
Synthesis of **3**.

**Scheme 2:**
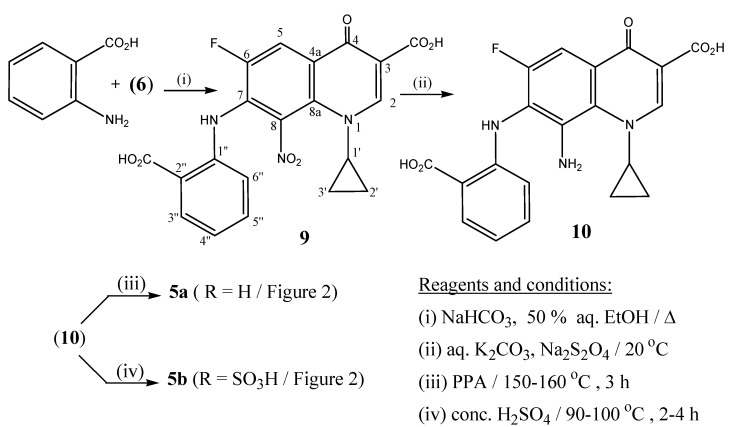
Synthesis of tetrahyro-1*H*-quino[7,8-*b*][1,4]benzodiazpine-3-carboxylic acid derivatives **5a**,**b**.

Owing to the potential biological interest in these heterocyclic compounds, the present research addresses the synthesis and characterization of new heterocyclic system incorporating 4-oxopyridine nucleus condensed either to 1,5-benzodiazpinone to form the target compound **3** (Figure 1, Scheme 1) or to the analogous dibenzo[*b*,*e*][1,4]Diazepinone to form compound **5a **(Figure 2, Scheme 2). Such hybrid tri- and tetracyclic systems (**3**, **5a**,**b**) might exhibit interesting bio-properties such as antimicrobial and/or antitumor activity.

## Results and Discussion

Preparation of the target benzodiazepine 2,8-dioxohexahydro-1*H*-[1,4]Diazepino[2,3-*h*]quinoline-9- carboxylic acid (**3**) was carried out *via* direct reaction of *β*-alanine with 7-chloro-1-cyclopropyl-6-fluoro-8-nitro-4-oxo-1,4-dihydroquinoline-3-carboxylic acid (**6**) in 50 % aqueous ethanol containing sodium bicarbonate (Scheme 1).

The primary amino group of *β*-alanine acts as a nucleophile that bonds to the C-7 of the quinolone nucleus by *via* a regiospecific nucleophilic aromatic substitution (addition-elimination) reaction. This mode of S_N_-Ar substitution reaction is mainly facilitated by the presence of the electron-withdrawing nitro group at C-8 of synthon **6**, together with the keto group and fluorine atom at positions 4 and 6, respectively.

Reduction of the 8-nitro derivative **7** with sodium dithionite in aqueous potassium carbonate furnished the respective 8-amino intermediate **8**. The latter underwent cyclization upon heating with polyphosphoric acid (PPA) or with concentrated sulphuric acid for 2-4 h to afford the tricyclic 2,8-dioxohexahydro-1*H*-[1,4]Diazepino[2,3-*h*]quinoline-9-carboxylic acid system (**3**), in high yields.

Similarly, interaction of 2-aminobenzoic acid with **6** provided the nitro derivative 7-[2-carboxyphenyl)amino]-1-cyclopropyl-6-fluoro-8-nitro-4-oxo-1,4-di-hydroquinoline-3-carboxylic acid (**9**, Scheme 2). Derivative **9** was then reduced with sodium dithionite to form the respective 8-amino derivative **10**. Lactamization of **10** using PPA gave the tetracyclic target product 1-cyclopropyl-6-fluoro-4,12-dioxo-4,7,12,13-tetrahydro-1*H*-quino[7,8-*b*][1,4]benzodiazepine-3-carboxylic acid (**5a**). In a separate step, compound **10 **underwent lactamization with sulfonation upon heating with concentrated sulphuric acid for 2-4 h, affording the dibenzodiazepine-10-sulphonic acid **5b**.

The identification of the prepared intermediates and target compounds was based on elemental analysis, IR, MS, ^1^H- and ^13^C-NMR spectral data, given in the Experimental. These spectral data were all consistent with the proposed structures. Signal assignments to the various proton and carbons were mostly determined following DEPT and 2D (COSY, HMQC and HMBC) experiments. It was clearly apparent that H-7 in **3** and H-5 in **5**, **7-10, **which resonate at around 8.0 ppm (d, ^3^*J*_H-F_ ≈ 13 Hz), showed consistent splitting patterns in all compounds due to coupling with the vicinal fluorine atom. It was revealed from the new broad signal at around 7 to 8 ppm, assigned for the NH at C-7, that the primary amine constituent was introduced in **7**, **9**. The same proton was down field shifted upon reduction of these compounds indicating the formation of the 8-amino derivative **8**, **10**. In case of the target compounds **3**, **5a**,**b** a singlet peak for the amide -NH was observed at around 10 ppm indicating that lactamization has taken place. For compound **3**, long-range correlations are observed between H-10 and each of C-8, C-11a and CO_2_H. Corresponding long-range correlations are also observed between H-7 and its neighbor carbons C-8, C-11a and C-5a. Similar pattern of long-range correlations were observed for **5a**,**b**. The skeletal carbons of the fused benzene ring (**B**) are recognizable by their signal splitting arising from coupling with fluorine atom (different value of *J* for each carbon) and from long-range coupling with neighboring protons.

### Antimicrobial activity

The *in vitro* antibacterial activity of all intermediates and targeted products was evaluated against an assortment of Gram positive and Gram negative bacterial strains using the minimum inhibitory concentration (MIC) approach. The prepared targets and the intermediates have shown interesting antibacterial activity mainly against Gram positive strains (Table 1), while none have shown any activity against Gram negative bacteria. The activity ranged from week to strong against both *S. aureus* (with MIC range 12.5-0.39 µg/mL) and *B. subtilis* (with MIC range 6.25-0.78 µg/mL). In particular, compound **8** showed good activity against *S. aureus* (with MIC 0.39 µg/mL) and *B. subtilis* (with MIC 0.78 µg/mL). It is generally assumed that the more lipophilic quinolones can penetrate better the lipophilic cell membrane of Gram positive bacteria, while less lipophilic compounds are more liable to penetrate the cell wall of Gram negative bacteria [52,53]. The activities of the target compounds (**3 **and **5**) and intermediates prepared in this work (**7-10**) are in correlation with this theory since they are lipophilic. On the other hand, the anthranilic acid derivatives **5a** and **9 **have also displayed excellent antifungal activity against *Candida albicans* with MIC values of 1.56 µg/mL and 0.78 µg/mL, respectively.

**Table 1:** MICs (*µ*g/mL) for compounds **3**, **5** and **7-10** against Gram positive bacterial strains and *Candida* *albicans.*

Compound No.	*S. aureus* ATCC 6538	*Bacillus subtilis ATCC 6633*	*Bacillus pumilus* *ATCC 8241*	*Candida albicans* *ATCC 1023*
**7**	ND*	6.25	ND	ND
**8**	0.39	0.78	6.25	ND
**3**	12.5	3.13	6.25	ND
**9**	0.78	6.25	3.13	0.78
**10**	3.13	ND	6.25	ND
**5a**	6.25	3.13	ND	1.56
**5b**	ND	1.56	6.25	ND
**Ciprofloxacin**	0.048	0.098	0.024	3.13

* ND: Not detected ( > 50 µg/mL )

### Cytotoxicity towards cancerous epithelial cells

Preliminary cytotoxicity studies were carried out for four candidate compounds (**3**, **8**, **5a**, **5b**) with MCF-7 cells, a human breast adenocarcinoma cell line, to test whether these compounds are toxic to epithelial cells, or they would have a potential as anticancerous agents. Cells were trypsinized, seeded in 96 well plates and incubated for 24 h. The substances were first dissolved in DMSO and then diluted with RPMI 1640 cell culture media, added to the cells, and incubated at a concentration range of 0.001 to 1.0 µg/mL. The cells were incubated with the compounds for 48 h, and sulphrodamine B assay was run afterwards. All tests were performed in triplicates and repeated twice using two different passages. All compounds did not change the proliferation rate of the cells as compared to controls (cells incubated with media only, with the same ratio of DMSO). This would suggest that these compounds are not toxic to epithelial cells. Further evaluation should be considered for exact determination of the IC_50_.

## Experimental

### General

2,4-Dichloro-5-fluoro-3-nitrobenzoic acid, ethyl 3-(dimethylamino)acrylate, *β*-alanine, cyclo- propylamine and 2-aminobenzoic acid were purchased from Acros. Melting points (uncorrected) were determined in open capillaries on a Stuart scientific electro-thermal melting point apparatus. Infrared (IR) spectra were recorded with Avatar Thermo Nicolet Impact 400 FT-IR spectrophotometer. Samples were prepared as potassium bromide discs. ^1^H- and ^13^C-NMR spectra were measured on a Varian 300 MHz spectrometer and a Bruker UltraShield-300 MHz instrument. Chemical shifts are given in *δ* (ppm) using tetramethylsilane (Me_4_Si) as internal reference and DMSO-d_6_ as solvent.

High resolution mass spectra (HRMS) were measured in negative ion mode by electrospray ionization (ESI) technique on a Bruker APEX-2 instrument. The samples were dissolved in acetone, diluted in spray solution (methanol + water + ammonia, in the ratio 1:1:1, v/v/v) and infused using a syringe pump with a flow rate of 2 mm^3^/min. External calibration was conducted using arginine cluster in a mass range *m/z* = 175-871.

Elemental analyses were performed on a Euro Vector Elemental Analyzer (EA 3000A-Italy). Thin layer chromatography (TLC) was performed on 10 x 10 cm^2^ aluminum plates pre-coated with fluorescent silica gel GF_254_ (ALBET, Germany). Mobile phase mixtures were chloroform: methanol: formic acid (95: 4: 1).

### 7-Chloro-1-cyclopropyl-6-fluoro-8-nitro-4-oxo-1,4-dihydroquinoline-3-carboxylic acid (***6***)

This compound was prepared from 2,4-dichloro-5-fluoro-3-nitrobenzoic acid and ethyl 3-(*N,N*-dimethylamino)acrylate, according to literature procedure [54,55,56,57].

### 7-[(2-Carboxyethyl)amino]-1-cyclopropyl-6-fluoro-8-nitro-4-oxo-1,4-dihydro-quinoline-3-carboxylic acid (*?**7***)

A stirred mixture of *β*-alanine (1.1 g, 12 mmol), synthon **6** (1.0 g, 3 mmol) and sodium hydrogen carbonate (1.5 g, 18 mmol) in 50 % aqueous ethanol (140 mL) was heated at 70-80 °C for 4-5 days under reflux conditions. The mixture slowly developed a light yellow color that changed into bright yellow, then into clear orange solution. The progress of the reaction was monitored by TLC, and was completed within 4-5days The mixture was extracted with dichloromethane (2 x 50 mL). The aqueous layer was cooled, its pH adjusted to 6-7 by addition of 3.5N HCl and re-extracted with CH_2_Cl_2_ (50 mL). Further acidification of the leftover aqueous layer to pH = 1-2 gave the title compound as yellowish solid which was collected by filtration, washed with cold water (2 x 10 mL), dried and re-crystallized from a mixture of chloroform and ethanol (1:1, v/v). Yield 1.0 g (88 %); mp 231–233 °C; *R_f_* value = 0.44; IR: *ν* 3522, 3400, 3363, 2927, 1715, 1628, 1549, 1511, 1423, 1318, 1238, 1213, 1124, 1075, 1034 cm^-1^; ^1^H-NMR: *δ* 0.93 (m, 4H, H_2_-2′/H_2_-3′), 2.57 (t, *J* = 6.5 Hz, 2H, C*H*_2_-CO_2_H), 3.66 (m, 1H, H-1′), 3.70 (br t, *J* = 6.5 Hz, 2H, NH-C*H*_2_), 7.39 (br t, *J* = 6.7 Hz, 1H, N*H*), 7.96 (d, ^3^*J*_H-F _= 14 Hz, 1H, H-5), 8.71 (s, 1H, H-2), 12.70 (br s, 1H, CH_2_-CO_2_*H*), 14.52 (br s, 1H, C (3)-CO_2_*H*); ^13^C- NMR: *δ* 10.2 (C-2°/C-3°), 35.3 (*C*H_2_-CO_2_H), 40.6 (C-1°), 42.2 (d, *J*_ C-F _= 12.9 Hz, *C*H2-NH), 109.5 (C-3), 114.7 (d, ^2^*J*_C-F _= 22.9 Hz, C-5), 116.5 (d, ^3^*J*_C-F _= 7.2 Hz, C-4a), 128.3 (d, ^3^*J*_C-F _= 5.5 Hz, C-8), 135.7 (C-8a), 138.8 (d, ^2^*J*_C-F _= 14.3 Hz, C-7), 150.4 (d, ^1^*J*_C-F _= 248 Hz, C-6), 151.9 (C-2), 165.4 (C(3)-*C*O_2_H), 173.3 (CH_2_*C*O_2_H), 175.4 (d, ^4^*J*_C-F _= 2.6 Hz, C-4); HRMS ((-ve)-ESI): *m/z* calcd. for C_16_H_13_FN_3_O_7_ [M-H]^−^: 378.07430, found: 378.07265; Anal. calcd. for C_16_H_14_FN_3_O_7_ (379.30): C, 50.67; H, 3.72; N, 11.08. Found: C, 50.94; H, 3.83; N, 11.31;

### 8-Amino-7-[(2-carboxyethyl)-amino]-1-cyclopropyl-6-fluoro-4-oxo-1,4-dihydro-quinoline-3-carboxylic acid (***8***)

To a stirred solution of compound **7** (0.38 g, 1 mmol) and potassium carbonate (0.96 g, 7 mmol) in water (20 mL) was added dropwise an aqueous solution of sodium dithionite (0.87 g, 5 mmol) in water (5 mL). The reaction mixture was further stirred at rt for 25 min. Thereafter, the pH of the solution was adjusted to about 4. The precipitated product was filtered, washed with water, air-dried and re-crystallized from acetone and ethanol (1:1, v/v) to furnish faint yellow crystals. Yield 0.32 g (92 %); mp 286-288 °C (decomp); *R_f_* value = 0.33; IR: *ν* 3514, 3373, 3333, 2917, 2724, 2662, 2587, 2530, 1730, 1677, 1591, 1536, 1448, 1418, 1334, 1269, 1199, 1151, 1074, 1030 cm^-1^; ^1^H-NMR: *δ* 1.01, 1.16 (2m, 4H, H_2_-2′/H_2_-3′), 2.52 (t, *J* = 6.5 Hz, 2H, C*H*_2_-CO_2_H), 3.33 (br t, *J* = 6.5 Hz, 2H, NH-C*H*_2_), 4.49 (m, 1H, H-1′), 5.02 (br s, 1H, N*H*), 5.55 (br s, 2H, N*H*_2_), 7.28 (d, ^3^*J*_H-F _= 11.2 Hz, 1H, H-5), 8.64 (s, 1H, H-2), 12.28 (br s, 1H, CH_2_-CO_2_*H*), 15.15 (br s, 1H, C(3)-CO_2_*H*); ^13^C-NMR: *δ* 10.6 (C-2°/C-3°), 35.1 (*C*H_2_-CO_2_H), 39.8 (C-1°), 41.8 (d, *J*_C-F _= 4.7 Hz, *C*H_2_-NH), 99.6 (d, ^2^*J*_C-F _= 23.6 Hz, C-5), 106.1 (C-3), 121.1 (d, ^3^*J*_C-F _= 9.2 Hz, C-4a), 128.1 (C-8a), 129.6 (d, ^2^*J*_C-F _= 15.5 Hz, C-7), 133.6 (d, ^3^*J*_C-F _= 5.9 Hz, C-8), 150.9 (C-2), 153.9 (d, ^1^*J*_C-F _= 239 Hz, C-6), 166.5 (C(3)-*C*O_2_H), 174.0 (CH_2_*C*O_2_H), 177.2 (d, ^4^*J*_C-F _= 3.2 Hz, C-4); HRMS ((-ve)-ESI): *m/z* calcd. for C_16_H_15_FN_3_O_5_ [M-H]^−^ : 348.10012, found: 348.10017; Anal. calcd. for C_16_H_16_FN_3_O_5_ (349.31): C, 55.01; H, 4.62; N, 12.03. Found: C, 54.78; H, 4.32; N, 11.95;

### 11-Cyclopropyl-2,8-dioxo-6-fluoro-2,3,4,5,8,11-hexahydro-1H-[1,4]Diazepino[2,3-h]quinoline-9-carboxylic acid (***3***)

Method (A)**:** A stirred solution of compound **8** (0.20 g, 0.57 mmol) and conc. sulphuric acid (8 mL) was heated at 100°C under reflux conditions for 3-5 h. The reaction mixture was then cooled to rt, and poured slowly onto ice (30 g). The precipitated product was then collected by suction filtration, washed with water (20 mL) and dried to furnish a brown-yellowish solid product. Yield 0.175 g (92 %); mp 346-347 °C (decomp); *R_f_* value = 0.38; IR: *ν* 3441, 2994, 2908, 1659, 1435, 1405, 1312, 1020, 956, 871cm^-1^; ^1^H-NMR: *δ* 0.94, 1.01 (2m, 4H, H_2_-2′/H_2_-3′), 2.74 (br t, *J* = 4.8 Hz, 2H, 2H-3), 3.79 (m, 2H, 2H-4), 4.20 (m, 1H, H-1′), 6.85 (d, *J* = 2.1 Hz, 1H, N(5)-*H*), 7.70 (d, ^3^*J*_H-F _= 11.1 Hz, 1H, H-7), 8.65 (s, 1H, H-10), 9.51 (s, 1H, N(1)-*H*), 15.10 (br s, 1H, CO_2_*H*); ^13^C-NMR: *δ* 9.6 (C-2°/C-3°), 34.1 (C-3), 41.0 (C-1°), 46.7 (C-4), 106.7 (d, ^2^*J*_C-F _= 21.5 Hz, C-7), 107.6 (C-9), 113.6 (d, ^3^*J*_C-F _= 5.0 Hz, C-11b), 116.6 (d, ^3^*J*_C-F _= 8.3 Hz, C-7a), 135.2 (C-11a), 138.3 (d, ^2^*J*_C-F _= 14.3 Hz, C-5a), 151.3 (C-10), 154.2 (d, ^1^*J*_C-F _= 242 Hz, C-6), 166.5 (C(9)-*C*O_2_H), 172.7 (C(2)), 176.5 (C-8); HRMS ((-ve)-ESI): *m/z* calcd. for C_16_H_13_FN_3_O_4_ [M-H]^−^: 330.08956, found: 330.09002; Anal. calcd. for C_16_H_14_FN_3_O_4_ (331.30): C, 58.01; H, 4.26; N, 12.68. Found: C, 58.12; H, 4.42; N, 12.37;

Method (B)**:** A stirred solution of compound **8** (0.2 g, 0.57 mmol) in polyphosphoric acid (PPA, 10 mL) was heated under reflux conditions (150-160 °C) for 3-4 h. The mixture was then cooled to 50 °C, and poured onto cold water (60 mL) with vigorous stirring. The precipitated light brown product was collected by suction filtration, washed with water (2 x 10 mL) and dried. Yield 0.18 g (95 %). This product showed identical spectral properties to a sample of **3** prepared by method (A) above.

### 7-[2-Carboxyphenyl)amino]-1-cyclopropyl-6-fluoro-8-nitro-4-oxo-1,4-dihydro-quinoline-3-carboxylic acid (***9***)

A stirred mixture of 2-aminobenzoic acid (3.8 g, 9 mmol), synthon **6** (1.0 g, 3 mmol) and sodium hydrogen carbonate (1.5 g, 18 mmol) in 50 % aqueous ethanol (140 mL) was heated at 70-75 °C for 6-7 days under reflux conditions. Work-up of the resulting reaction mixture as described for **7 **above, gave the title compound as dark yellow solid. Yield 1.18 g (92 %); mp 292–294 °C ; *R_f_* value = 0.71; IR: *ν* 3437, 3068, 1745, 1669, 1616, 1550, 1514, 1445, 1402, 1312, 1246, 1158, 1108, 1028 cm^-1^; ^1^H- NMR: *δ* 0.97, 1.08 (2m, 4H, H_2_-2′/H_2_-3′), 3.74 (m, 1H, H_-_1′), 6.88 (dd, *J* = 7.41, 7.34 Hz, 1H, H-6′′), 7.03 (dd, *J* = 7.52, 7.50 Hz, 1H, H-4′′), 7.44 (dd, *J* = 7.48, 7.45 Hz, 1H, H-5′′), 7.92 (d, *J* = 7.45 Hz, 1H, H-3′′), 8.28 (d, ^3^*J*_H-F _= 11.17 Hz, 1H, H-5), 8.83 (s, 1H, H-2), 10.45 (br s, 1H, N*H*-Ar), 13.65 (br s, 1H, Ar-CO_2_*H*), 14.25 (br s, 1H, C(3)-CO_2_*H,* overlapping with Ar-CO_2_*H*); ^13^C-NMR: *δ* 10.6 (C-2°/C-3°), 39.5 (C-1°), 109.6 (C-3), 115.7 (d, ^2^*J*_C-F _= 21.6 Hz, C-5), 115.8 (d, overlapping with C-5, C-4a), 117.2 (d, *J*= 5.6 Hz, C-6°′), 121.9 (C-4°′), 123.3 (d, ^3^*J*_C-F _= 7.35 Hz, C-8), 130.2 (d, ^2^*J*_C-F _= 16.6 Hz, C-7), 131.6 (C-5°′), 133.5 (C-8a), 134.4 (C-3°′), 137.2 (C-2°′), 143.6 (d, *J* = 2.1 Hz, C-1°′), 153.0 (C-2), 153.1 (d, ^1^*J*_C-F _= 253 Hz, C-6), 165.1 (C(3)-*C*O_2_H), 170.1 (Ar-*C*O_2_H), 175.8 (d, ^4^*J*_C-F _= 2.0 Hz, C-4); HRMS ((-ve)-ESI): *m/z* calcd. for C_20_H_13_FN_3_O_7_ [M-H]^−^ : 426.07430, found: 426.07355; Anal. calcd. for C_20_H_14_FN_3_O_7_ (427.34): C, 56.21; H, 3.30; N, 9.83. Found: C, 56.14; H, 3.13; N, 9.50;

### 8-Amino-7-(2-carboxy-phenylamino)-1-cyclopropyl-6-fluoro-4-oxo-1,4-dihydro-quinoline-3-carboxylic acid (***10***)

To a stirred solution of compound **9** (0.43 g, 1 mmol) and potassium carbonate (0.96 g, 7 mmol) in 20 ml water was added dropwise an aqueous solution of sodium dithionite (0.87 g, 5 mmol) in water (5 mL). The reaction mixture was further stirred at rt for 30 min. Thereafter, the pH of the solution was adjusted to about 4 and the precipitated product was collected by filtration, washed with water, air-dried and re-crystallized from acetone and ethanol (1:1, v/v) producing faint yellow crystals of **10**. Yield 0.29 g (73 %); mp 287–289 °C; *R_f_* value = 0.50; IR: *ν* 3488, 3392, 2924, 2366, 1719, 1673, 1591, 1551, 1502, 1450, 1326, 1243, 1155, 1083, 1044 cm^-1^; ^1^H-NMR: *δ* 1.20 (m, 4H, H_2_-2′/H_2_-3′), 4.56 (m, 1H, H_-_1′), 5.93 (br s, 2H, NH_2_), 6.40 (d, *J* = 9.0 Hz, 1H, H-6′′), 6.78 (dd, *J* = 9.0, 6.0 Hz, 1H, H-4′′), 7.29 (dd, *J* = 6.0, 3.0 Hz, 1H, H-3′′), 7.35 (d, ^3^*J*_H-F _= 9.0 Hz, 1H, H-5), 7.93 (dd, *J* = 9.0, 3.0 Hz, 1H, H-5′′), 8.77 (s, 1H, H-2), 9.75 (br s, 1H, N*H*-Ar), 14.31 (br s, 1H, C(3)-CO_2_*H*), 15.05 (br s, 1H, C(2)-CO_2_*H,* overlapping with Ar-CO_2_*H*); ^13^C-NMR: *δ* 10.63 (C-2°/C-3°), 39.7 (C-1°), 97.9 (d, ^2^*J*_C-F _= 23.0 Hz, C-5), 106.8 (C-3), 113.6 (C-6°′), 117.9 (C-4°′), 119.0 (d, ^2^*J*_C-F _= 16.5 Hz, C-7), 122.0 (C-8), 126.0 (d, ^3^*J*_C-F _= 9.8 Hz, C-4a), 127.7 (C-8a), 131.9 (C-3°′), 133.9 (C-5°′), 140.4 (d, *J* = 3.7 Hz, C-2°′), 147.6 (C-1°′), 151.2 (C-2), 157.1 (d, ^1^*J*_C-F _= 243 Hz, C-6), 166.2 (C(3)-*C*O_2_H), 170.8 (C-(2°′)-*C*O_2_H), 177.3 (d, ^4^*J*_C-F _= 3.0 Hz, C-4); HRMS ((-ve)-ESI): *m/z* calcd. for C_20_H_15_FN_3_O_5_ [M-H]^−^ : 396.10012, found: 396.10097; Anal. calcd. for C_20_H_16_FN_3_O_5_ (397.36): C, 60.45; H, 4.06; N, 10.57. Found: C, 60.53; H, 3.86; N, 10.35;

### 1-Cyclopropyl-6-fluoro-4,12-dioxo-4,7,12,13-tetrahydro-1H-quino[7,8-b][1,4]benzodiazepine-3-carboxylic acid (***5a***)

A stirred solution of compound **10** (0.2 g, 0.5 mmol) and PPA (10 mL) was heated under reflux conditions (150-160 °C) for 3 h. The resulting mixture was then cooled to 50 °C, and poured onto cold water (60 mL) with vigorous stirring. The precipitated yellowish green solid product was collected by suction filtration, washed with water (2 x 10 mL) and dried. Yield 0.18 g (95 %); mp 325–326 °C (decomp); *R_f_* value = 0.63; IR: *ν* 3433, 2994, 2909, 2585, 2315, 2222, 2099, 1659, 1435, 1412, 1312, 1026, 957, 702, 671 cm^-1^; ^1^H-NMR: *δ* 0.85, 1.08 (2m, 4H, H_2_-2′/ H_2_-3′), 4.31 (m, 1H, H-1′), 7.10 (dd, *J* = 7.5, 7.5 Hz, 1H, H-10), 7.27 (d, *J* = 7.8 Hz, 1H, H-8), 7.44 (ddd, *J* = 7.2, 6.9, 2.1 Hz, 1H, H-9), 7.73 (dd, *J* = 6.9, 1.9 Hz, 1H, H-11), 7.82 (d, ^3^*J*_H-F_ = 10.2 Hz, 1H, H-5), 8.63 (d, *J* = 2.4 Hz, 1H, N(7)-*H*), 8.74 (s, 1H, H-2), 10.03 (br s, 1H, N(13)-*H*)), 15.20 (br s, 1H, CO_2_*H*); ^13^C-NMR: *δ* 9.9 (C-2°/C-3°), 41.2 (C-1°), 107.6 (d, ^2^*J*_C-F_ = 21.0 Hz, C-5), 108.1 (C-3), 120.6 (d, ^3^*J*_C-F_ = 3.9 Hz, C-13a), 121.9 (d, ^3^*J*_C-F_ = 7.2 Hz,C-4a), 122.0 (C-13b), 123.7 (C-10), 125.3 (C-8), 132.3 (C-9), 133.9 (C-11), 134.3 (C-11a), 141.1 (d, ^2^*J*_C-F_ = 15.9 Hz, C-6a), 149.5 (C-7a), 151.4 (d, ^1^*J*_C-F_ = 245 Hz, C-6), 152.0 (C-2), 166.1 (C(3)-CO2H), 168.9 (C-12), 176.9 (d, ^4^*J*_C-F_ = 2.7 Hz, C-4); HRMS ((-ve)-ESI): *m/z* calcd. for C_20_H_13_FN_3_O_4_ [M-H]^−^ : 378.08956, found: 378.08925; Anal. calcd. for C_20_H_14_FN_3_O_4_ (379.34): C, 63.32; H, 3.72; N, 11.08. Found: C, 63.45; H, 3.65; N, 10.96.

### 1-Cyclopropyl-6-fluoro-4,12-dioxo-10-sulfo-4,7,12,13-tetrahydro-1H-quino[7,8-b][1,4]benzo-diazepine-3-carboxylic acid (***5b***)

A stirred solution of compound **10** (0.2 g, 0.5 mmol) and conc. sulphuric acid (8 mL) was heated under reflux conditions (100 °C) for 3 h. The resulting mixture was poured onto water (60 mL) with vigorous stirring. The precipitated solid product was collected by suction filtration, washed with water (2 x 10 mL) and dried to furnish green-yellowish solid product. Yield 0.17 (74%); mp 297–299 °C; *R_f_* value = 0.60; IR: *ν* 3433, 2994, 2909, 1651, 1435, 1408, 1312, 1057, 1026, 957, 903 cm^-1^; ^1^H-NMR: *δ* 0.87, 1.09 (2m, 4H, H_2_-2′/ H_2_-3′), 4.31 (m, 1H, H_-_1′), 7.20 (d, *J* = 8.7 Hz, 1H, H-8), 7.61 (dd, *J* = 8.4, 2.1 Hz, 1H, H-9), 7.81 (d, ^3^*J*_H-F _= 10.0 Hz, 1H, H-5), 7.96 (d, *J* = 1.8 Hz, 1H, H-11), 8.70 (d, *J* = 2.7 Hz, 1H, N(7)-*H*), 8.72 (s, 1H, H-2), 10.02 (br s, 1H, N(13)-*H*)), 14.42-15.32 (br s, 2H, CO_2_*H +* SO_3_*H*); ^13^C-NMR: *δ* 9.9 (C-2°/C-3°), 40.5 (C-1°), 107.6 (d, ^2^*J*_C-F _= 22.0 Hz, C-5), 108.2 (C-3), 120.6 (d, ^3^*J*_C-F _= 3.8 Hz, C-13a), 121.4 (C-8), 122.1 (d, ^3^*J*_C-F _= 7.2 Hz,C-4a), 124.0 (C-10), 129.6 (C-9), 131.2 (C-11), 134.3 (C-13a), 134.4 (C-11a), 140.6 (d, ^2^*J*_C-F _= 16.0 Hz, C-6a), 149.4 (C-7a), 151.4 (d, ^1^*J*_C-F _= 246 Hz, C-6), 152.1 (C-2), 166.1 (C(3)-*C*O_2_H), 168.6 (C-12), 176.9 (d, ^4^*J*_C-F _= 2.7 Hz, C-4); HRMS ((-ve)-ESI): *m/z* calcd. for C_20_H_13_FN_3_O_7_S [M-H]^−^: 458.04637, found: 458.04661; Anal calcd. for C_20_H_14_FN_3_O_7_S (459.41): C, 52.29; H, 3.07; N, 9.15. Found: C, 52.16; H, 2.98; N, 9.02.

### In vitro antibacterial activity testing

Nutrient agar and Nutrient broth were obtained from Himedia, Mumbai, India. 0.5 McFarland suspension was prepared by adding BaCl_2_ (1.175 % w/v BaCl_2_∙2H_2_O, 0.5 mL) to 0.36 N H_2_SO_4_ (1.0 % v/v, 99.5 mL). Sterilization of materials and equipments was carried out using Raypa steam sterilizer Autoclave. Microbiology samples were incubated at 37 °C using WTC binder incubator. 96-Flat bottom microplates were used in the conduction of broth dilution test. ELx 800 UV universal microplate reader, Biotek instrument was used to determine the turbidity in the wells. Ciprofloxacin.HCl was used as reference. The bacterial strains used were *Escherichia coli* ATCC 8731 and *Staphylococcus aureus* ATCC 6538, resistant isolates from both *E. coli* and *Staph. aureus*, *Proteus valgaris* spp. ATCC 7542, *Bacillus subtilis* ATCC 6633, *Bacillus pumilus* ATCC 8241 and *Candida albicans* ATCC 1023. Bacterial suspensions were prepared in sterilized distilled water, in a concentration around 1x10^7^ cfu/mL, which was standardized according to 0.5 McFarland suspension as described by the Clinical and Laboratories Standards Institute (CLSI) 2007. The minimum inhibitory concentrations (MICs, µg/mL) of test compounds were determined by broth dilution method, screening different concentrations in the range 100–0.097 µg/mL. The MIC is defined as the lowest concentration of the tested compound showing no growth. A stock solution of each tested compound was prepared in DMSO (100 µg/mL). The MIC test was performed in 96 flat bottom microtiter plates, 100 µL of previously prepared and sterilized broth (prepared by dissolving 1.3 g of dry preparation in 100 mL of distilled water) was added in each well, with an exception to the first well where 100 µL of double strength, sterilized broth was added (prepared by dissolving 1.3 g of dry preparation in 50 mL of distilled water) in order to maintain the consistency of the broth along the plate after the addition of the tested compound. An equivalent volume of 50 µg/mL of each compound was added to the first well, mixed with the broth, followed by two fold serial dilution onto successive wells across the plate to end up with 11 successive two fold dilutions for each of the tested compounds. Then 10 µL of bacterial suspension was used to inoculate each well. Control tests for each experiment were performed. Positive growth control was performed by adding one drop of each micro-organism suspensions to four wells in each plate of the culture medium without the test compound. Negative growth control was also performed using four un-inoculated wells of medium without the test compound. Plates were incubated at 37 °C for 24 h, and were checked for turbidity. Two fold serial dilutions were carried out in a similar manner for DMSO (20% v/v in water) to test its antibacterial activity. Ciprofloxacin standard was tested also as reference compound. The turbidity was determined visually and using microplate reader.
